# Effect of Acetamiprid, a Neonicotinoid Insecticide, on Locomotor Activity of the American Cockroach

**DOI:** 10.3390/insects15010054

**Published:** 2024-01-12

**Authors:** Emiliane Taillebois, Alison Cartereau, Steeve H. Thany

**Affiliations:** Laboratory Physiology, Ecology and Environment (P2E), University of Orleans, USC-INRAE 1328, 1 rue de Chartres, 45067 Orleans, France; emiliane.taillebois@univ-orleans.fr (E.T.); alison.cartereau@univ-orleans.fr (A.C.)

**Keywords:** insect, cockroach, locomotor activity, insecticide, neonicotinoid, acetamiprid

## Abstract

**Simple Summary:**

Acetamiprid, which is a neonicotinoid, is a cyano-substituted insecticide. It is commonly used due to its rapid insecticidal activity. Acetamiprid has been described as a partial agonist of nicotinic acetylcholine receptors expressed on the thoracic and dorsal neurons of the American cockroach. However, previous electrophysiological studies have demonstrated that not all neonicotinoids display the same modes of action on insects. In this study, we analysed the effect of acetamiprid on the locomotor activity of the American cockroach and compared our results with previously published data for thiamethoxam and clothianidin, which are nitro-substituted neonicotinoid insecticides. We demonstrated that the three neonicotinoids had different effects on the locomotor activity. Overall, these data add to the understanding of the mode of action of neonicotinoid insecticides.

**Abstract:**

Toxicological studies have shown that the American cockroach *Periplaneta americana* (Linnaeus) is a classical model for studying the mode of action of commonly used insecticides. In a previous study, we demonstrated that thiamethoxam and clothianidin decreased locomotor activity in an open-field-like apparatus. Here, we tested the effect of the neonicotinoid acetamiprid when applied orally, topically, or injected into the haemolymph. We found that acetamiprid was also able to impair locomotor activity in the open-field-like apparatus. When treated with acetamiprid, a strong alteration in locomotor activity was observed 1 h, 24 h, and 48 h after haemolymph and topical applications. Oral application induced an impairment of locomotor activity at 24 h and 48 h. A comparison of the present data with our previously published results showed that neonicotinoids were more active when injected into the haemolymph compared to oral and topical applications. These findings increased our understanding of the effect of neonicotinoid insecticides on insect locomotor activity, and demonstrated that the cyano-substituted neonicotinoid, acetamiprid, was able to alter cockroach locomotor activity.

## 1. Introduction

The American cockroach *Periplaneta americana* (L.) is an insect pest in urban entomology, a potential vector of various pathogenic organisms, and also produces allergens that are responsible for allergies and asthma [1,2]. It is currently used as a model for studying the mode of action of several compounds, including neonicotinoid insecticides [3,4,5,6,7]. Neonicotinoids are agrochemical compounds, which were discovered in the 1980s [8]. They are strongly efficient in controlling sap-feeding insects in agriculture [9,10]. The seven major commercial neonicotinoids are divided into three structural compounds including chloropyridinyl (imidacloprid, nitempyram, thiacloprid, and acetamiprid), clorothiazolyl (clothianidin and thiamethoxam), and tetrahydrofuryl (dinotefuran) [8,11]. Currently, their mode of action is thought to be related to their capacity to bind to nicotinic acetylcholine receptors as agonists [12,13,14,15,16]. Neonicotinoids were split into two different subgroups, depending on their action on *P. americana* thoracic neurons [7]. Those with a heterocyclic ring in their electronegative pharmacophore moiety (imidacloprid and thiacloprid) and the open chain compounds (acetamiprid, dinotefuran, nitenpyram, and clothianidin), which were much more effective agonists compared to acetylcholine [7]. Moreover, different neonicotinoids also caused two distinct types of intoxication, which were associated with their chemical structures. For example, imidacloprid and thiacloprid, which have a heterocyclic electronegative moiety, caused strong excitatory symptoms, with uncoordinated quivering, hyper-excitability, and rapid spontaneous movements. By contrast, the open chain compounds elicited different poisoning symptoms. In this group, acetamiprid was the exception, causing excitation symptoms rather than depression and paralysis [7]. Acetamiprid is formed with chloropyridinylmethyl and N-cyanoimine groups [17]. In a recent study comparing dinotefuran, imidacloprid, and acetamiprid, it was demonstrated that acetamiprid and dinotefuran had a stronger hydrogen-bond acceptor site than imidacloprid due to their cyano and nitro moieties, respectively [18]. The crystal structure of acetamiprid pointed out cooperative π–π and hydrogen bond interactions important for binding with nicotinic acetylcholine receptors [18]. Electrophysiological studies performed on cockroach dorsal unpaired median (DUM) neurons, located in the sixth abdominal ganglion, showed that a pressure application of acetamiprid onto isolated DUM neuron somata induced a biphasic dose–response curve. These results demonstrated that it acted as an agonist on both cockroach nicotinic acetylcholine receptor 1 and 2 subtypes, whereas imidacloprid only activated the subtype 1 [19]. At the synaptic level, using the synapse between the cercal afferent giant interneuron and the nerve XI, it was demonstrated that acetamiprid induced a strong dose-dependent increase in the ganglionic depolarisation [18]. Thus, acetamiprid was able to activate synaptic and extra-synaptic nicotinic acetylcholine receptor subtypes.

**Figure 1:**
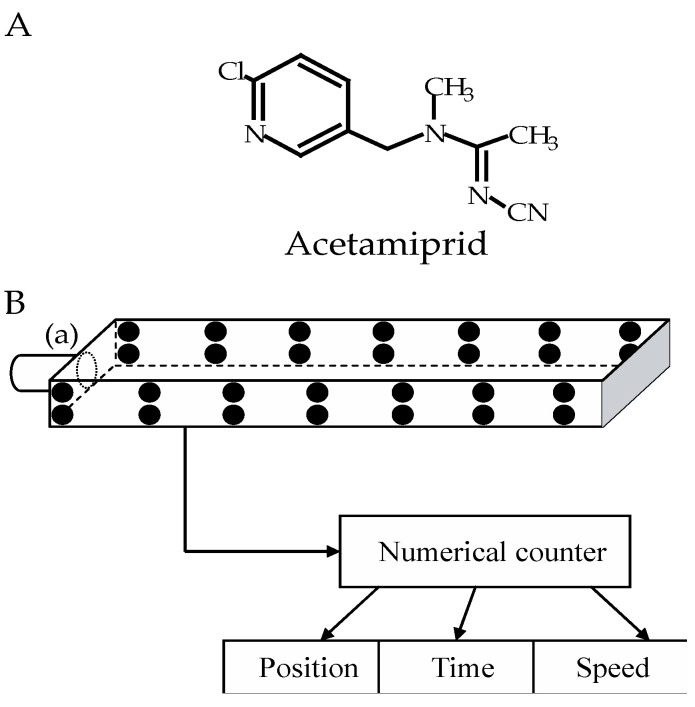
(**A**) Structure of the chloropyridylmethyl compound acetamiprid. (**B**) Schematic representation of the open-field-like apparatus; 28 infrared light-emitting diodes (black circles) enable the detection of cockroaches’ position through the device. At the beginning of the experiment, the tested cockroach was placed in the cylinder (a) and blocked by closed trap door in this starting area. When the door opens, the cockroach’s movements were recording continuously using the numerical counter, allowing for its position, speed, and the duration of immobility to be determined.

In the present study, we explored the effect of acetamiprid (Figure 1A) on *P. americana* locomotor activity and discussed the results regarding our previous studies on the effects of other neonicotinoids. Indeed, using an open-field-like apparatus (Figure 1B), we demonstrated that at higher doses (0.5 nmol.g^−^^1^ and 1 nmol.g^−^^1^), clothianidin led to uncoordinated movements, leg shakings, and prostration in intoxicated cockroaches. However, when exposed to thiamethoxam, cockroaches only demonstrated excitatory signs with a characteristic shaking of legs [19]. Moreover, we found that the two major nitro-imines (thiamethoxam [20] and clothianidin [21]) decreased cockroach locomotor activity. The percentage of cockroaches displaying locomotor activity was significantly reduced one hour after haemolymph application. No significant effect was found after topical and oral administration of thiamethoxam and clothianidin. Thiamethoxam levels remained persistent 48 h after application, and the amount of clothianidin in cockroach tissues was persistent with the toxicity of thiamethoxam [22]. The main conclusion was that the effect of thiamethoxam was partly due to its metabolization into clothianidin. Here, we studied the effect of the cyano-imine acetamiprid using the same experimental conditions.

## 2. Materials and Methods

### 2.1. Animals

Cockroaches *P. americana* used for the experiments were obtained from Arbiotech (Saint Gilles, France). Until use, they were kept in aquaria at 29 °C, under a 12 h:12 h dark:light cycle, and with food (large breed adult mature dog, HILLS, France) and water provided ad libitum. Before acetamiprid administration, cockroaches were anesthetized on ice (4 °C) for 10 min [23]. Then, all experiments were performed at room temperature. The experimental procedures were in compliance with the European Laws (86/609/CEE) on the use of animals.

### 2.2. Chemicals

All chemicals were purchased from Sigma-Aldrich (Saint Quentin, France). Acetamiprid technical grade insecticide (CAS: 135410-20-7, purity ≥ 98%) was purchased from Sigma Aldrich (France) (Figure 1A) and was dissolved in dimethylsulfoxide (DMSO) to prepare a concentrated stock solution. The stock solution was extemporaneously diluted to obtain a working solution of acetamiprid at 0.01% DMSO. The control consisted of DMSO diluted to a final concentration of 0.01% DMSO in a saline solution. The saline solution contained (in mM): NaCl 208; KCl 3.1; CaCl_2_ 5.4; NaHCO_3_ 2; sucrose 26; and had a pH of 7.4 [24].

### 2.3. Exposure Protocol

Acetamiprid was applied to cockroaches in three different ways at concentrations of 0.1, 0.5, and 1 nmol.g^−1^ [22]. For topical application, 1 µL of the final solution was applied to the thorax (dorsal surface) of the cockroach using a Hamilton syringe. As previously described, for the haemolymph treatment, 10 µL of solution was injected between the third and fourth sternites [25]. Oral application consisted of a starvation for the day before the experiment, and then each cockroach was fed with 5 µL solution (either acetamiprid or control solution) [22]. For each condition, acetamiprid was administered to the cockroaches one time the day before the experiment.

### 2.4. Evaluation of Locomotor Activity

The locomotor activity of cockroaches was individually evaluated using an open-field-like apparatus (32 × 6.5 × 3 cm), according to the method used by Lambin et al. [26], with slight adaptations of the device. Two series of 14 LEDs divided the device into seven different levels and allowed for the observation of cockroaches’ horizontal displacements (Figure 1B). The cockroaches were tested 1 hour after acetamiprid or control exposure. Each individual was introduced to the open-field-like apparatus, via the cylinder (a), which remained closed by the trap door [22]. At the beginning of the recording, the door opened and the cockroach walked from the left to the right freely for 5 min. Each cockroach was recorded one time in the open-field-like apparatus. Several parameters such as the position and the crossing time of the device (average time between the trap door and the last LED) were automatically scored. The proportion of cockroaches displaying locomotor activity were evaluated 1 h, 24 h, and 48 h after application of acetamiprid or control solutions.

### 2.5. Evaluation of Cockroaches’ Mortality

To evaluate the direct toxicity of acetamiprid, the total number of dead cockroaches was determined for each acetamiprid concentration (0.1, 0.5, and 1 nmol.g^−1^) applied either by injection into the haemolymph, topical application, or oral application. The mortality was scored at 1 h, 24 h, and 48 h after acetamiprid exposure. The number of dead cockroaches observed in the corresponding control condition was used to calculate the corrected mortality percentage, using Henderson Tilton’s formula [27].

### 2.6. Mannitol-Gap Recordings

Mannitol-gap experiments were performed according to the previously published protocol [28,29,30,31]. Cockroaches were dissected and opened along the longitudinal dorsal-median line with a fine pair of forceps. The sixth abdominal ganglion, nerve cord, one circus, and the corresponding cercal nerve XI were isolated in saline solution containing (in mM) NaCl, 208; KCl, 3.1; CaCl_2_, 5.4; NaHCO_3_, 2; Sucrose, 26; pH 7.4 [24,30,31]. The preparation was transferred to the recording chamber and continuously superfused with mannitol solution (87 g.L^−1^). The excitatory postsynaptic potentials and action potentials were evoked using electrical stimulation of cercal nerve XI with a dual-pulse stimulator (Campden Instruments, Loughborough, UK). The recording electrodes were connected to a high-impedance amplifier, of which the output was passed to a digital oscilloscope, a chart recorder, and a computer for off-line analysis. Data were analysed using an analogue-to-digital converting interface (Hameg, Mainhausen, Germany). Data were analysed using PClamp 10 software (Axon Instruments, Foster City, CA, USA) [24,30,31]. Data are the mean of at least five recordings.

### 2.7. Statistical Analysis

We used a set of simulation scenarios to estimate the probability of detecting a significant difference between the acetamiprid and control groups (Using R. Program). A generalized linear model (GLM, R Program) with repeated measures to compare three factors was adopted: concentration (0.1, 0.5, and 1 nmol.g^−1^), application (haemolymph, topical, and oral), and treatment (acetamiprid and control), using R [32]. The variable was the binomial family in which ‘1’ corresponded to a cockroach eliciting locomotor activity and ‘0’ to a cockroach without locomotor activity. We also used Student *t*-test and Welch’s correction to illustrate the relationship between control and treated cockroaches (GraphPad Prism 9, Boston, MA, USA, www.graphpad.com (accessed on 4 November 2023)).

## 3. Results

### 3.1. Percentage of Cockroaches Displaying Locomotor Activity

**Figure 2:**
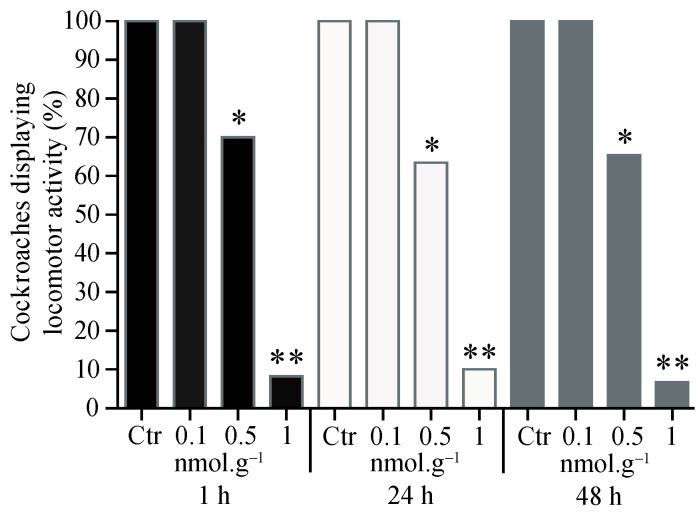
Proportion of cockroaches eliciting locomotor activity 1 h, 24 h, and 48 h after acetamiprid injection into the haemolymph. For each condition, 20 cockroaches were tested. Control group (Ctr). Significant differences between acetamiprid treatment and control, using Student *t*-test, are indicated with asterisks (* *p* < 0.05 and ** *p* < 0.001).

Over the 180 cockroaches tested, a significant difference appeared between each application (χ^2^ = 44.48, *p* < 0.001), concentration (χ^2^ = 38.50, *p* < 0.001), and treatment (χ^2^ = 22.27, *p* < 0.001). The proportion of cockroaches eliciting locomotor activity decreased under haemolymph application, after 1 h, for the following doses, 0.5 nmol.g*^−^*^1^ (t = 96.0, df = 4, *p* < 0.05, *n* = 20) and 1 nmol.g*^−^*^1^ (t = 1105, df = 4, *p* < 0.001, *n* = 20, Figure 2), compared to the control group. The decrease in the locomotor activity was also found for the same doses at 24 h (0.5 nmol.g*^−^*^1^: t = 39.1, df = 4, *p* < 0.05, *n* = 20; 1 nmol.g*^−^*^1^: t = 724.8, df = 4, *p* < 0.001, *n* = 20) and 48 h (0.5 nmol.g*^−^*^1^: t = 60.9, df = 4, *p* < 0.05, *n* = 20; 1 nmol.g*^−^*^1^: t = 226.2, df = 4, *n* = 20) after exposure to acetamiprid.

**Figure 3:**
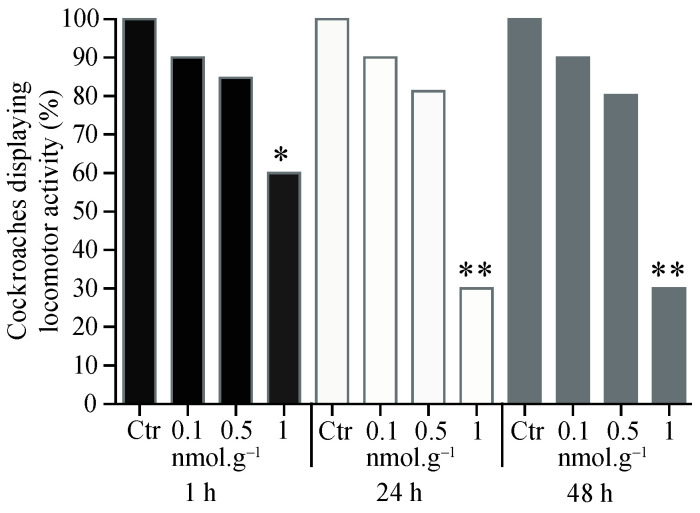
Proportion of cockroaches eliciting locomotor activity 1 h, 24 h, and 48 h after topical application of acetamiprid. For each condition, 20 cockroaches were tested. Control group (Ctr). Significant differences between acetamiprid treatment and control, using Student *t*-test, are indicated with asterisks (* *p* < 0.05 and ** *p* < 0.001).

For the topical application of acetamiprid (Figure 3), a decrease in locomotor activity was only found at concentrations of 1 nmol.g*^−^*^1^ and was observed at all time delays, 1 h (t = 36.3, df = 4, *p* < 0.05, *n* = 20), 24 h (t = 43.5, df = 4, *p* < 0.001, *n* = 20), and 48 h (t = 43.4, df = 4, *p* < 0.05, *n* = 20).

**Figure 4:**
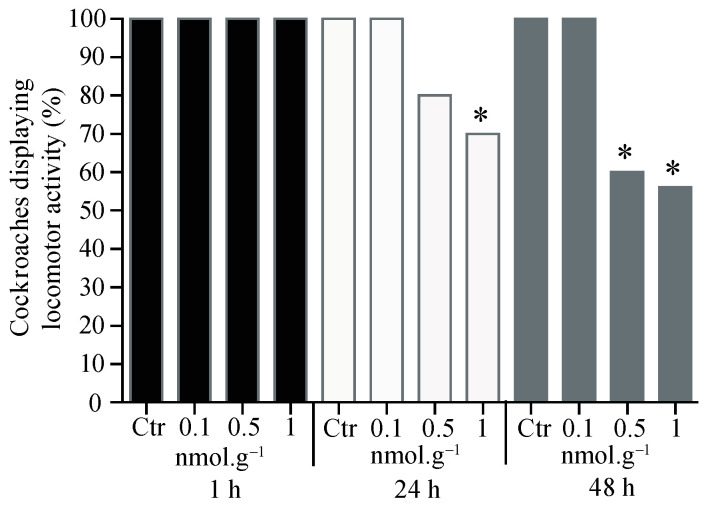
Proportion of cockroaches eliciting locomotor activity after 1 h, 24 h, and 48 h oral application of acetamiprid. For each condition, 20 cockroaches were tested. Control group (Ctr). Significant differences between acetamiprid treatment and control, using Student *t*-test, are indicated with asterisks (* *p* < 0.05).

Oral application of acetamiprid led to a significant lowering of locomotor activity at 0.5 nmol.g*^−^*^1^ for 48 h (t = 46.5, df = 4, *p* < 0.05, *n* = 20). Similarly, a decrease was also found at 1 nmol.g*^−^*^1^ at 24 h (t = 30.2, df = 4, *p* < 0.05, *n* = 20) and 48 h (t = 78.0, df = 4, *p* < 0.05, *n* = 20) (Figure 4).

### 3.2. Cockroach Mortality after Acetamiprid Application

We assessed the direct toxicity of acetamiprid for the different tested concentrations and for each acetamiprid exposure method. The mortality scores correspond to corrected mortality percentage (with *n* = number of cockroaches tested). No mortality was observed at 1 h after exposure to acetamiprid for the different concentrations (0.1, 0.5, and 1 nmol.g*^−^*^1^) and exposure methods (haemolymph, topical, or oral application). At 24 h, no mortality was found after topical application of acetamiprid at the different concentrations. After haemolymph injection, acetamiprid led to 5% mortality at 0.5 nmol.g*^−^*^1^ and 1 nmol.g*^−^*^1^; for both concentrations we did not observe any mortality compared to the corresponding control conditions (*p* > 0.05, *n* = 50). We did not observe any dead individuals at the lowest concentration (0.1 nmol.g*^−^*^1^). At this delay, an increase in mortality was found in orally intoxicated cockroaches. In particular, oral application of acetamiprid at 1 nmol.g*^−^*^1^ induced 10% mortality at 24 h, although this percentage was not statistically different from the control condition (*p* > 0.05, *n* = 50). The lower concentrations (0.1 and 0.5 nmol.g*^−^*^1^) did not induce any mortality 24 h after oral exposure.

**Figure 5:**
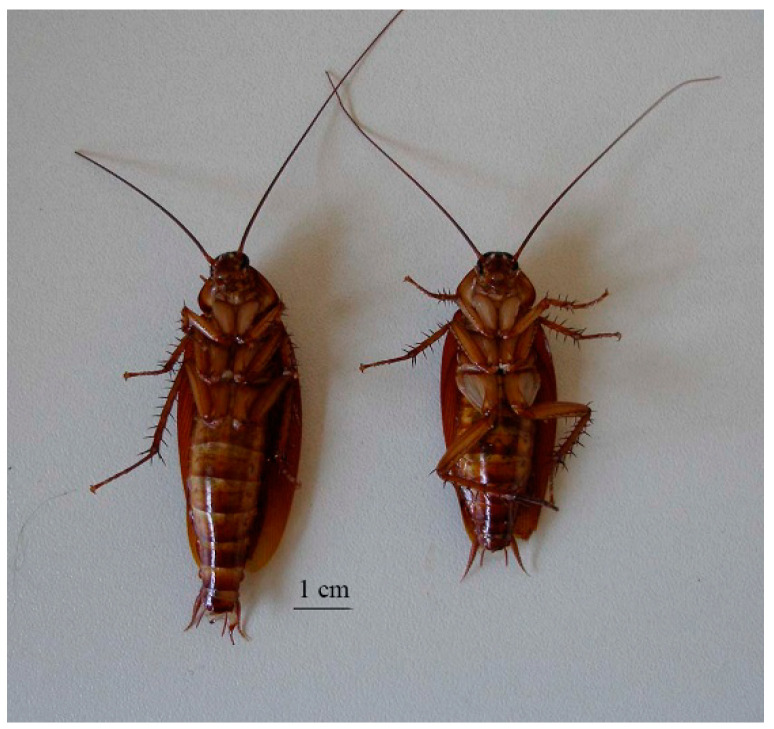
Illustration of the morphological aspect of cockroaches after 1 nmol.g^−^^1^ haemolymph application of acetamiprid (on the left) compared to untreated cockroach (on the right).

At 48 h after acetamiprid application, no mortality was observed for haemolymph, topical, or oral applications of 0.1 nmol.g*^−^*^1^. However, for haemolymph application, a significant toxicity was observed with 20% mortality at 0.5 nmol.g*^−^*^1^ (*p* < 0.05, *n* = 50) and 35% mortality at 1 nmol.g*^−^*^1^ (*p* < 0.05, *n* = 20). A similar concentration-dependent toxicity was observed 48 h after topical application, with 10% (*p* > 0.05, *n* = 50) and 20% (*p* < 0.05, *n* = 50) mortality at 0.5 nmol.g*^−^*^1^ and 1 nmol.g*^−^*^1^, respectively. We also found that acetamiprid applied orally induced a significant toxicity with 35% mortality at a concentration of 1 nmol.g*^−^*^1^ (*p* < 0.05, *n* = 50 cockroaches), whereas no mortality was observed at 0.5 nmol.g*^−^*^1^. In addition, we observed some particularities concerning cockroaches that were more sensitive and thus died after application of acetamiprid. We first found that they were unable to walk normally, and they presented an increase in their body length compared to control cockroaches. The body length was measured at the end of the experiments. Normal and treated cockroaches were between 3.5–4 cm and 5.5–6 cm in length, respectively (Figure 5). Note that the maximum length was observed in dead cockroaches.

### 3.3. Characterization of Cockroach Locomotor Activity: Immobility and Time of Exploration

**Figure 6:**
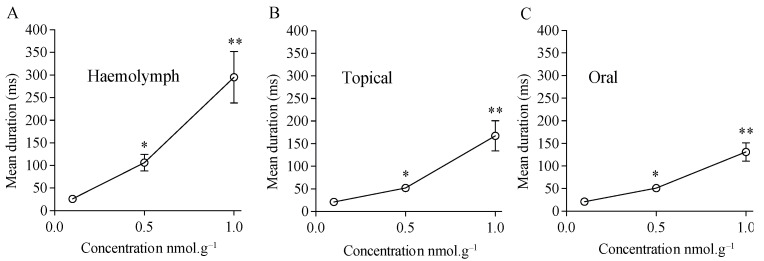
Mean duration of immobility of cockroaches after acetamiprid treatment. The duration of immobility was measured during 5 min of observation in the open-field-like apparatus. Recordings were obtained 1 h after acetamiprid exposure through, (**A**) haemolymph, (**B**) topical, and (**C**) oral applications. Significant differences between treated and control conditions are marked with asterisks (* *p* < 0.05, ** *p* < 0.01, Student *t*-test with Welch’s correction).

For the cockroaches displaying locomotor activity, we evaluated the locomotor behaviour more precisely. In particular, we determined the duration of immobility and the time of exploration. These measurements were performed 1 h after acetamiprid application to avoid any bias due to direct toxicological effect. We observed that the duration of immobility strongly increased when acetamiprid was applied at higher concentrations (Figure 6). Indeed, no significant difference was found with control groups at 0.1 nmol.g*^−^*^1^ after haemolymph (t = 1.14, df = 85.9, *p* > 0.05, *n* = 50), topical (t = 1.52, df = 90.3, *p* > 0.05, *n* = 50), and oral (t = 0.65, df = 90.3, *p* > 0.05, *n* = 50) applications. After exposure to acetamiprid at 0.5 nmol-g*^−^*^1^, a significant difference was revealed between untreated and treated cockroaches with an exposure through haemolymph (t = 5.4, df = 51.2, *p* < 0.05), topical (t = 5.9, df = 63.2, *p* < 0.05, *n* = 50), or oral (t = 6.5, df = 68, *p* < 0.05, *n* = 50) applications. At 1 nmol.g*^−^*^1^, a significant difference was found after haemolymph (t = 5.0, df = 49.2, *p* < 0.05, *n* = 50), topical (t = 4.8, df = 49.6, *p* < 0.05, *n* = 50), and oral (t = 6.1, df = 50.7, *p* < 0.05, *n* = 50) applications.

To assess the exploration speed of the cockroaches, we measured the time needed for each individual from the trap door of the cylinder (starting area) to the last LED of the open-field-like apparatus. The average time needed to cross the experimental device is reported in Table 1 for the different methods of exposure and the different concentrations of acetamiprid. We found that the time of exploration was increased in a similar way to the immobility duration. In fact, the time needed to reach the last LED was increased at higher concentration (0.5 and 1 nmol.g^−1^) but was not modified at 0.1 nmol.g^−1^.

### 3.4. Effect of Acetamiprid on Cockroach Sixth Abdominal Ganglion

To complete our study, we aimed to evaluate if excitatory postsynaptic potentials or action potentials could be recorded in treated cockroaches that presented a decrease in locomotor activity. We focused our study on cockroaches treated with 1 nmol.g^−^^1^ acetamiprid after haemolymph injection, and topical or oral applications because a major effect on locomotor activity was found with 1 nmol.g^−^^1^. Thus, using mannitol-gap recording, the sixth abdominal ganglion from treated cockroaches were compared as performed in our previous studies [18].

**Figure 7:**
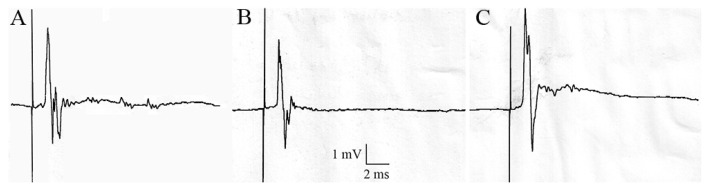
Mannitol-gap recording studies. Electrical stimulation of nerve XI on treated cockroaches with 1 nmol.g^−^^1^ acetamiprid through haemolymph injection (**A**), topical (**B**), and oral (**C**) applications. Electrical stimulations of the nerve XI were recorded 20 min after locomotor activity in the open-field-like apparatus.

Our results showed that the sixth abdominal ganglia from cockroaches treated with 1 nmol.g^−^^1^ acetamiprid presented action potentials similar to control cockroaches after electrical stimulation of nerve XI (Figure 7). Thus, in control cockroaches, the electrical stimulation led to action potentials of 3.82 ± 0.10 mV, whereas they were 3.21 ± 0.14 mV (*n* = 6 ganglia, Figure 7A), 3.20 ± 0.16 mV (*n* = 5, Figure 7B), and 3.93 ± 0.10 mV (*n* = 6, Figure 7C) after haemolymph, topical, and oral applications of 1 nmol.g^−^^1^ acetamiprid. These results suggest that synaptic transmission in cockroaches, which showed a decrease in locomotor activity, was not affected by acetamiprid.

## 4. Discussion

The present study focused on the effect of acetamiprid on the locomotor activity of *Periplaneta americana*. Our results show an effect of concentration and application methods of acetamiprid on the locomotor activity. We first found that at higher concentrations, the mean duration of immobility increased irrespective of the application method. A similar effect has been previously found with thiamethoxam and clothianidin [22], confirming that at higher doses, exposure to neonicotinoids increased the mean duration of immobility in the open-field-like apparatus. Indeed, we showed that when acetamiprid was injected to the haemolymph or applied topically, a decrease in locomotor activity was found after application, whereas no effect was seen after oral administration. A strong effect occurred at higher concentrations for all application methods. Similar discrepancies were found in our previous study using thiamethoxam and clothianidin [22]. This suggests that the three neonicotinoids altered the locomotor activity differently, depending on the application method. Indeed, a comparison of the present data with our previously published results showed that neonicotinoids were more active when injected into the haemolymph compared to oral and topical applications. We proposed that acetamiprid is more active than clothianidin and thiamethoxam one hour after application. Similar analysis performed at 24 h showed a significant effect when thiamethoxam was applied topically. We also highlighted a concentration-dependent effect of acetamiprid, which was more effective at higher concentrations. Moreover, we found that despite its effect on locomotor activity, acetamiprid did not affect synaptic transmission in the treated cockroaches. Using mannitol-gap recording, we found that electrical stimulation of the nerve XI, which is the largest and the most posterior of the nerves in the sixth ganglion, induced excitatory postsynaptic potentials and action potentials. We propose that the underlying circuitry of cockroach locomotor activity affected by acetamiprid is independent to sensitive interneurons in the abdominal nerve cord, in particular giant interneurons in the sixth abdominal ganglion. Indeed, for insecticides there are several potential sites of action located at the presynaptic terminal and/or on the postsynaptic side. We also noted that acetamiprid affected cockroaches’ length. We are unable to explain the potential molecular mechanisms of this change, which does not seem specific to neonicotinoids. In a recent study, it was demonstrated that zebrafish embryos exposed to varying concentrations of (-)-R-indoxacarb, a pro-insecticide, exhibited a reduction in body length [33]. We hypothesized that acetamiprid could induce exoskeletal muscles relaxation, in particular within the muscle connecting the tergites or the connections of the tergites, leading to changes cockroach length after exposure. The effect of neonicotinoid insecticides on locomotor activity was previously demonstrated in the honeybee *Apis mellifera* using an open-field apparatus [34]. In naive honeybees, it was demonstrated that neonicotinoids increase the time spent immobile, and the distance covered was reduced in treated honeybees [35]. Moreover, thiamethoxam and acetamiprid had no significant effect on the parameters of locomotor activity of treated honeybees compared to control groups. The same lack of effect was observed whatever the method used to apply the neonicotinoids [34]. This result was in accordance with our observations. The most significant finding in our studies was that, as with acetamiprid and clothianidin, thiamethoxam altered cockroach locomotor activity, despite the fact that we did not observe uncoordinated movements or leg tremors. Consequently, we confirmed that thiamethoxam had its own effect, which was not associated with its metabolite clothianidin as previously proposed [35]. In this previous experiment, one explanation could be that thiamethoxam and clothianidin were differently metabolized in cockroaches and/or had a different action on their targets. This explanation is consistent with the finding that the metabolism of neonicotinoid either increases or decreases its potency depending on the compound and specificity of the nicotinic acetylcholine receptor subtypes [7,8,21,36,37]. Acetamiprid is a neonicotinoid with a chloropyridinyl group and a carboxamidine that is acetamidine, in which the amino hydrogens are substituted by a (6-chloropyridin-3-yl)methyl and a methyl group while the hydrogen attached to the amino nitrogen is replaced by a cyano group. Based on the chemical structure of neonicotinoids, recent studies have demonstrated that cytochrome P450 enzymes efficiently detoxified N-cyanoamidine compounds with little activity against N-nitroguanidine compounds [38,39,40]. For example, an increased tolerance to thiacloprid and acetamiprid was found in transgenic *Drosophila melanogaster* expressing the *CYP9Q6* gene. Drosophila expressing the CYP9Q6 transgene were approximately 2.3 times more resistant to acetamiprid [40]. The link between neonicotinoids and cockroach nicotinic receptors has been demonstrated in several studies [41,42,43,44,45]. Neonicotinoid agonist actions vary significantly, including partial, full, and super activities. Clothianidin, which has an acyclic moiety corresponding to the imidazolidine moiety of imidacloprid, was referred to as a super or full agonist [7,46], and acetamiprid was the least efficacious analog in the acyclic group [7]. These agonist activities appeared to be related both to their structure and the cockroach nicotinic acetylcholine receptor subtypes expressed in the insect’s central nervous system [7]. These discrepancies between the three neonicotinoids were also observed in other studies. Several nicotinic acetylcholine receptor subtypes have been identified between the cercal afferent giant interneuron synapses and in the fast coxal depressor neurons [24,47]. Clothianidin, acetamiprid, and thiamethoxam acted differently as agonists of the cockroach nicotinic receptors expressed in the ventral nerve cord [7,48]. Given this background information, we suggested that neonicotinoids which alter the cholinergic neurons through their action on insect neuronal nicotinic acetylcholine receptors could also impair locomotor behaviour of *P. americana*.

## 5. Conclusions

In the present study, we demonstrated that the cyano-substituted neonicotinoid, acetamiprid, which differently acted as an agonist of cockroach nicotinic acetylcholine receptors, could also impair locomotor activity, as found with the nitro-substituted thiamethoxam and clothianidin.

## Figures and Tables

**Table 1:** Time needed for the cockroaches to cross the experimental device 1 h post acetamiprid treatment. For each exposure method, untreated cockroaches (control group) received the saline solution in the same experimental condition as the treated cockroaches (see Materials and methods).

Exposure Method	Control	Acetamiprid (nmol.g^−1^)		
		0.1	0.5	1
Haemolymph injection	6.2 ± 0.7 s	6.4 ± 1.7 s	129.3 ± 25.0 s	319.0 ± 67.0 s
Topical application	5.9 ± 2.1 s	6.4 ± 2.5 s	74.4 ± 2.0 s	189.0 ± 3.0 s
Oral application	6.1 ± 1.7 s	6.0 ± 2.0 s	73.4 ± 17.0 s	137.7 ± 38.0 s

## Data Availability

Data presented in this study are available on request from the corresponding author.
